# Research on the Visualization of Railway Signal Operation and Maintenance Based on BIM + GIS

**DOI:** 10.3390/s23135984

**Published:** 2023-06-28

**Authors:** Yanming Liu, Haixiang Lin, Zhengxiang Zhao, Wansheng Bai, Nana Hu

**Affiliations:** 1 State Key Laboratory of Rail Transit Engineering Informatization, Xi’an 710043, China; zzx511229689@163.com; 2 School of Automation and Electrical Engineering, Lanzhou Jiaotong University, Lanzhou 730070, China; 19193156561@163.com (Z.Z.); bws199904@163.com (W.B.); 17339820212@163.com (N.H.)

**Keywords:** railway signal, BIM, GIS, dynamic scheduling, three-dimensional visualization

## Abstract

To adapt to the “fine” and “extensive” management characteristics of railway signal equipment operation and maintenance, achieving real-time and interactive monitoring of signal equipment operation status, and developing an integrated approach to equipment operation and maintenance, this paper takes a comprehensive management perspective. To create a lightweight BIM model, the Garland folding algorithm is utilized to simplify the IFC file format. Building on this approach, the data are divided based on building component division standards to obtain separate files containing geometric information and semantic attributes. The geometric information files are converted to a 3D Tiles format, combining BIM semantic attributes with semantic attribute files through an intermediate format. Dynamic data management is achieved by setting the octree space index structure in combination with a view-frustum culling algorithm. Then, the 3D Tiles target file is imported into the Cesium platform, and Node.js is used to achieve three-dimensional visualization of railway signal operation and maintenance. The proposed method is verified using an inbound signal as an example to assess its feasibility. The results demonstrate the potential of the proposed method to achieve stable integration between BIM equipment full lifecycle maintenance and GIS geographical space display. Railway signal equipment is endowed with comprehensive one-click information query functions for equipment positioning and spatial analysis, improving the efficiency and scientific decision-making level of equipment operation and maintenance.

## 1. Introduction

As society progresses and the economy grows, the scale of railway construction in China continues to expand, entering a peak period of operation and maintenance. Presently, on-site railway maintenance is still based on the operation and maintenance concept of relying on experience rather than science [1]. Manual inspection is central to evaluating and maintaining signal equipment, resulting in low operational and maintenance efficiency, high subjectivity, and poor collaboration in real time. Obtaining a horizontal coupling relationship between system equipment through mechanism or big data analysis presents a challenge, making it challenging to address the severe challenges of high-speed railway operation and maintenance [2]. Digital twin technology [3], a dynamic digital technology that reflects the entire lifecycle of entity systems, integrates physical models, data transmission, information sharing, and other methods to simulate the multidimensional visual simulation process of railway equipment operation and maintenance, providing valuable support for intelligent operation and maintenance information integration and sharing services. Collecting and integrating equipment information in railway engineering forms the fundamental basis of digital twin technology. Accurately constructing the digital twin model of equipment information represents a critical step in the railway digital twin system, allowing for the achievement of virtual–real interaction [4]. To achieve digital twins in railways, the integral technologies of building information modeling (BIM) and geographical information systems (GIS) represent a pivotal aspect [5].

BIM models are capable of achieving digital virtualization for various buildings and equipment through precise geometric structures and rich semantic information. The key characteristic of BIM is its ability to fully display equipment models and support the storage and management of the equipment’s full lifecycle information [6]. Railway signal engineering involves multiple disciplines, has a wide range of applications, and involves complex maintenance technology. By integrating BIM technology into the management system, the degree of information sharing can be quickly improved, and equipment management control can be optimized, resulting in the further enhancement of the system’s digital visualization and intelligence [7,8]. However, in signal equipment management, equipment is relatively dispersed, has a wide installation area, and there is a shortage of maintenance personnel with only limited maintenance time. Moreover, even if specific equipment information is presented, it cannot keep up with the real-time geographical location and operational status of the equipment for the workers. Consequently, workers cannot view large-scale models, which results in the inconsistent analysis of the signal equipment system [9].

GIS, on the other hand, is more capable of visualizing large-scale scenes and spatial analysis of the geographic area. Unlike BIM, GIS focuses more on displaying macro-environmental information and spatial models of equipment’s external area [10]. Combining BIM and GIS, all-round management of equipment maintenance can be achieved to address the management requirements for “precise” signal equipment and “extensive” work areas. Furthermore, this will solve the problem of GIS’s simple 3D model information content and the independent separation of BIM models. The integration of the full lifecycle of equipment, geographic environmental data, construction information, and maintenance engineering information is achieved, leading to the comprehensive integration of spatial and temporal information to form an organically unified visual model [11]. This integration will enable multiple functions like equipment positioning, spatial analysis, and information inquiry relating to the maintenance, alarm prediction, and emergency response of signal equipment. This process will improve the system’s scientific decision-making level. However, there are still challenges that exist in the actual application process of BIM + GIS technology.

BIM and GIS represent different industries that cater to different objects; hence, their models differ [12]. Regarding space characteristics, the BIM design relies on local coordinate systems, while the GIS design is based on global coordinate systems. BIM models contain not only geometric and appearance information but also valuable semantic information. In contrast, GIS provides effective data management for supporting geospatial analysis, but it lacks complete physical model information. The difference between BIM and GIS requires BIM models to be converted before directly integrating with GIS platforms while preserving the semantic information in the BIM models. The integration of BIM and GIS has gained attention and has been applied across various professional fields [13,14]. Although the integration of BIM–GIS can extend model information, semantic interoperability between BIM and GIS should also be ensured. For example, Sharafat et al. [15] analyzed the advantages and disadvantages of BIM–GIS conversion from the perspective of semantic and geometric information and proposed an optimization method for data exchange between BIM and GIS datasets. In their research on integrated BIM and GIS solutions, Liu and Borrmann [16,17] devised a data model to facilitate their integration. Critical issues in the BIM–GIS integration process includes improving loading and querying efficiency and addressing model format conversion. Zhao, Zhu, and Tang [18,19,20] proposed a method for converting semantic-constrained Revit model format RVT into CityGML model format by mapping IFC format files to different levels of CityGML. Zhao and Zhang [21,22] developed an intelligent railway construction and management information system based on GIS and BIM technologies to provide decision-making support for railway construction management. Peng [23] integrated typical Revit geometry and semantic information into 3DGIS, along with spatial and dynamic construction data generated during railway construction. Although these methods provide a basis for integrating railway signal equipment with BIM and GIS, the efficiency of using WebGL technology for graphic rendering using GIS through a client’s GPU and memory resources on the web is affected by low network bandwidth and server performance, leading to reduced efficiency in loading and applying models [24,25]. Railway signal equipment has complicated types and delicate components, and although software tools can convert BIM data to GIS data, they are limited to less detailed models. The relationship between equipment types at different levels of detail (level of detail, LOD) [26] is unclear, and the degree of coupling with semantic information is low. Therefore, efficient BIM–GIS methods for integrating railway signal equipment must be developed to improve the operation and maintenance efficiency of railway signal equipment.

This paper aims to address the challenges associated with signal equipment by exploring their full lifecycle management based on a digital twin. This study concentrates on monitoring the health status of equipment, pinpointing faults accurately, converting BIM model data, and dynamically scheduling spatial data, which serve as the research content. We propose an approach to integrating large-scale GIS and BIM datasets on the web and offer a feasible solution. Additionally, we developed a BIM + GIS-based railway signal operation and maintenance visualization platform. The platform provides real-time and interactive monitoring of the system’s operation status, which aims to enhance the intelligent operation and maintenance of railway signal equipment.

## 2. Research Framework

**Figure 1:**
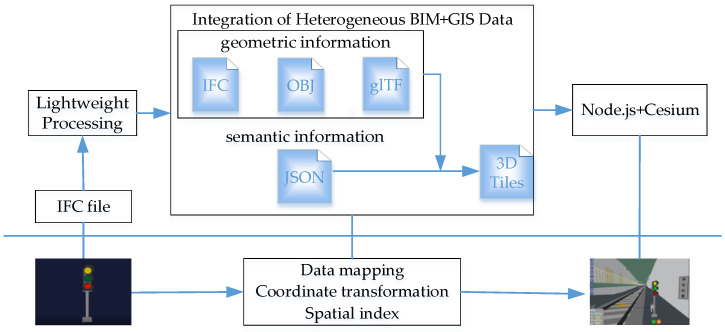
The research framework of railway signal operation and maintenance visualization based on BIM + GIS.

This article takes the railway signal BIM model as the research object. First, the IFC standard file is exported from Revit. After that, the BIM model is simplified, and the file format is converted. The geometric properties are stored as glTF files, and the corresponding semantic properties are saved in a JSON file. Based on this, the glTF file and JSON file are packaged to obtain a b3dm-type 3D tile to realize the transfer of model attribute information to 3D Tiles while completing the data mapping, coordinate conversion, and spatial indexing. Eventually, the target format of 3D Tiles is loaded into the Cesium engine, and with the help of Node.js, the 3D visualization needs of railway signal operation and maintenance are catered for in the integrated platform. The study framework is displayed in Figure 1.

### 2.1. Lightweight Processing

Model lightweighting refers to the use of algorithms to reduce a model’s data volume while retaining its shape integrity, allowing for a faster and more convenient display. Geometric information is a crucial component of a model’s data and determines the memory space occupied by the model file. By simplifying the geometric information of models, their overall volume can be significantly reduced, enabling faster transmission speed. Lightweight processing also offers the only means of realizing models across multiple or cross-platforms. As such, this paper will focus on the grid information that makes up the geometric entity of the model and simplify it in order to achieve a lighter-weight operation of the model.

The models’ geometric information is stored in triangular mesh grids. While software can quickly simplify the geometric structure of models, the current simplification process only targets specific surface algorithms, disregarding the need for verifying surface expressions. Complex BIM models often need millions of triangular meshes to depict their characteristics, and transmitting and rendering such a massive amount of data presents a significant challenge on the web. Thus, this study uses the Garland edge-folding algorithm based on quadratic-error measurements provided by the Revit API to simplify the triangular mesh grid.

The fundamental concept involves using edges as the fundamental geometric elements to be removed. When an edge is folded, a new point is produced, while all points linked to the removed edge are connected to the new point to retain the surface of the model as a triangular mesh grid. By controlling the sequence and number of edge folds, models with different resolutions can be obtained.

Define the error value $\Delta \bar{v}$ between any vertex $v={\left[x,y,z,1\right]}^{T}$ and the new vertex $\bar{v}$ in the mesh, where Δv is the sum of the square distances from the individual triangles in the set of associated triangles, Planes(v), containing $\bar{v}$ to a plane, as shown in Equation (1).

(1) $$\Delta \left(v\right)=\sum_{p\in Planes\left(v\right)}\left(p^{T}v\right)$$

In this equation, $p={\left[a,b,c,d\right]}^{T}$ denotes the plane equation for each triangle found in Planes(v) and is represented by ax+by+cz+d=0, alongside $a^{2}+b^{2}+c^{2}=0$.

After a simple matrix transformation, the following can be obtained:

(2) $$\Delta \left(v\right)=\sum_{p\in Planes\left(v\right)}{\left(p^{T}v\right)}^{2}=\sum_{p\in Planes\left(v\right)}v^{T}pp^{T}v=v^{T}\left(\sum_{p\in Planes\left(v\right)}K_{p}\right)v.$$

One of the key components in our study is the matrix Kp, which is a four-by-four symmetric matrix. This matrix can be described as the error matrix of the triangle, and its definition is as follows:

(3) $$K_{p}=pp^{T}=\left(\begin{array}{cccc} a^{2} & ab & ac & ad\\ ab & b^{2} & bc & bd\\ ac & bc & c^{2} & cd\\ ad & bd & cd & d^{2} \end{array}\right).$$

The steps that make up the quadratic error-based simplification algorithm are as follows in detail: ① input the vertex sequence, which includes the xyz coordinates of each vertex and the triangle face sequence, containing the vertex numbers of each triangle; ② calculate the normal vector of each face; ③ calculate the plane coefficient $ax+by+cz+d=0,p=\left[a,b,c,d\right]$ for each face; ④ calculate the initial error matrix, “$K_{p0}$” for each vertex by computing $pp^{T}$ for each vertex; ⑤ compute the error matrix “Q” for each vertex, which is the sum of the fundamental error matrix, “$K_{p}$” associated with each plane.

During the algorithm operation, it is crucial to prevent the occurrence of thin triangles and the inconsistent behavior of mesh flipping. Prior to folding each edge, pre-judgment is made to check whether thin triangles will occur or not. If so, edge folding is not performed. Formula (4) presents the exact calculation.

(4) $$C=\frac{4\sqrt{3}a}{l_{0}^{2}+l_{1}^{2}+l_{2}^{2}}$$

In the formula, $l_{0}$, $l_{1}$, and $l_{2}$ represent the three sides’ length of a triangle, and a is its area. A triangle is equilateral when C=1 and degenerates into a line when C=0.

**Figure 2:**
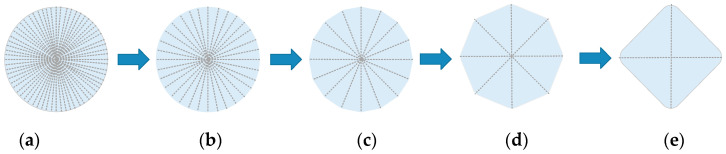
The simplification effect of mesh information. (**a**) LOD5; (**b**) LOD4; (**c**) LOD3; (**d**) LOD2; (**e**) LOD1.

The Garland edge fold procedure, which is based on the quadratic error metric, produces a uniform distribution for the simplified model mesh. The model’s detail level can be determined as needed, which allows for obtaining models with different degrees of detail. Figure 2 illustrates the simplification result.

### 2.2. Integration of Heterogeneous BIM + GIS Data

The industries and target services of BIM and GIS are distinct, resulting in divergent approaches to their respective models. GIS utilizes the earth’s coordinate system to represent geographical features. Conversely, BIM uses a local coordinate system. This difference in coordination systems impacts the interoperability of the two technologies. Furthermore, beyond geometry and appearance information, BIM also encompasses extensive semantic content. Given these disparities, the integration of BIM and GIS models is necessary for the consistent application of BIM data in large-scale scenes.

#### 2.2.1. Coordinate Transformation

**Figure 3:**
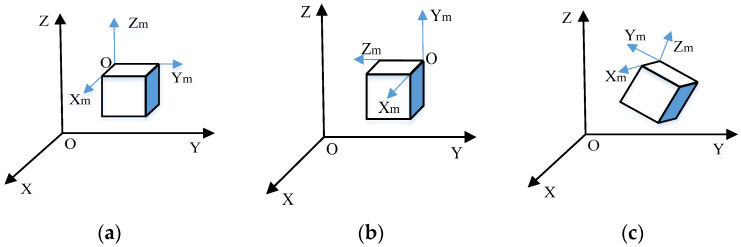
Comparison of coordinate systems. (**a**) Revit coordinate system; (**b**) glTF coordinate system; (**c**) WGS-84 coordinate system.

First, the unification of the coordinate systems between BIM and GIS is necessary. The BIM coordinate system maintains precise topological and positional relationships between all the elements of a complex model in space using a local coordinate system. Meanwhile, the GIS coordinate system, which is mostly based on the WGS-84 earth coordinate system, is shown in Figure 3. Zhao et al. [27] constructed basic industrial buildings, aligning the origin of the local coordinate system with the WGS-84 latitude and longitude coordinates. They also adjusted the model inclination angle to merge the two coordinate systems and retain semantic information within BIM models. Industrial basic building components and railway signal equipment components are divisible and independent. This shared characteristic inspired the current study to apply the method mentioned above to the railway field. To achieve a unified coordinate system, this paper sets the coordinate origin to the model benchmark point in the modeling software and uses the same value as that of the WGS-84 coordinate origin. By implementing a consistent transformation between the coordinates of the model benchmark point and the measured benchmark point, the coordinates of the former can be determined in GIS. The calculation method of the conversion matrix $T_{t}$ between the two coordinate systems can be expressed as follows:

(5) $$T_{t}=R_{x}R_{y}R_{z}T$$

In the above equation:

(6) $$R_{x}=\left[\begin{array}{cccc} 1 & 0 & 0 & 0\\ 0 & \cos\theta_{x} & \sin\theta_{x} & 0\\ 0 & -\sin\theta_{x} & \cos\theta_{x} & 0\\ 0 & 0 & 0 & 1 \end{array}\right]$$

(7) $$R_{y}=\left[\begin{array}{cccc} \cos\theta_{y} & 0 & -\sin\theta_{y} & 0\\ 0 & 1 & 0 & 0\\ \sin\theta_{y} & 0 & \cos\theta_{y} & 0\\ 0 & 0 & 0 & 1 \end{array}\right]$$

(8) $$R_{z}=\left[\begin{array}{cccc} 1 & 0 & 0 & 0\\ 0 & \cos\theta_{z} & \sin\theta_{z} & 0\\ 0 & -\sin\theta_{z} & \cos\theta_{z} & 0\\ 0 & 0 & 0 & 1 \end{array}\right]$$

(9) $$T=\left[\begin{array}{cccc} 1 & 0 & 0 & 0\\ 0 & 1 & 0 & 0\\ 0 & 0 & 1 & 0\\ x_{1} & y_{1} & z_{1} & 1 \end{array}\right].$$

The equation includes various components: $\left(x_{1},y_{1},z_{1}\right)$ denotes the position of the origin of the local coordinate system of the model in the WGS-84 coordinate system, which is based on the latitude and longitude of the location where the model is set; $R_{x}$, $R_{y}$, and $R_{z}$ represent the rotation matrices that correspond to each coordinate axis, respectively; $\theta_{x}$, $\theta_{y}$, and $\theta_{z}$ signify the rotation vectors specific to the angles between the rotation vector and the *X*, *Y*, and *Z* axes.

#### 2.2.2. File Format Conversion

After completing the coordinate system conversion, in order to improve the transfer, loading, and rendering efficiency of the railway signal equipment BIM model and enable the visualization and application functions of the railway signal equipment BIM three-dimensional model, it is necessary to convert the BIM model. The process of converting the BIM file format can be segmented into the following steps: IFC file generation, glTF file generation, and 3D Tiles file generation.

IFC file generation. IFC (industry foundation class) is a standard developed by Autodesk in 1994 to define a unified data format for expandable architectural information. To achieve uniformity in the railway signal equipment BIM model, various types of railway signal equipment component three-dimensional models are exported to IFC standard files in Revit. The IFC standard’s geometric entity provides a comprehensive description of railway signal equipment component shapes, sizes, and spatial positions in BIM models. However, the geometric information of railway equipment components in the IFC file is implicit, which requires further processing to convert this information to explicit geometric data that are suitable for rendering in the front end for visual three-dimensional model generation.

**Figure 4:**
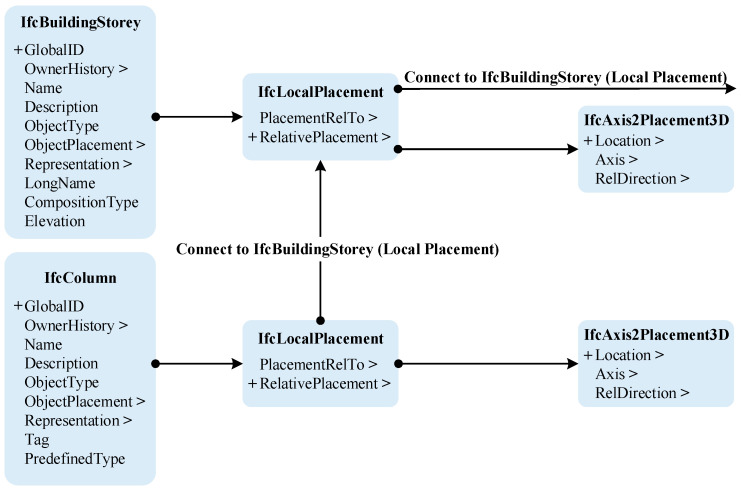
IFC file structure.

When developing a BIM model for railway signal equipment using the IFC standard, the container for all equipment components within the project must be defined in a single IfcProject. Components are differentiated by an IfcBuildingStorey, and their relationship is established based on different IfcBuildingStoreys, determining their hierarchy and component coordinate information in the 3D space. This relationship is achieved using IfcRelAggregates, defining hierarchical relationships between various spatial levels, with subspaces linked through the relationship object. All spatial elements are then related to IfcProject in this way. The BIM model process can establish a complete information model capable of covering information on IfcSite, IfcBuilding, IfcBuildingStorey, and IfcSpace elements through a set relationship. Figure 4 demonstrates the IFC file structure of a railway signal equipment component within an IfcBuildingStorey.

(1) Generation of glTF files. The first step in converting the IFC model representation is extracting the relevant information. We converted and exported the IFC file to the OBJ format using IfcConvert in IfcOpenShell. Subsequently, the OBJ file must be converted into a 3D model format to achieve the optimal 3D visualization and application of the BIM model. Poorly formatted 3D models may result in missing equipment components. To comply with the format requirements of the Cesium engine for good visualization effects, we chose glTF as the 3D model format for converting the OBJ file. The glTF file, after conversion, includes the following: specifically, the JSON file documents the entire file structure. Additionally, a binary (bin) file is included, storing specific data related to the respective model. Also incorporated is the GLSL file, containing shader information essential for the model’s rendering process.
(2) Generating 3D Tiles Files. The Cesium engine is a configurable viewer for glTF files with 3D models, structures, and textures, but it is only capable of displaying 3D visual elements. To display virtual representations such as equipment properties, additional access to a database or the use of form queries is necessary. However, the “3D Tiles” data standard effectively solves this problem, as it modularizes and stores 3D data hierarchically, lessening the burden on the web and CPU. This allows for the transmission of 3D data and large-scale rendering. The “3D Tiles” data standard has become a proprietary format for the WebGL-based Cesium engine.

**Figure 5:**
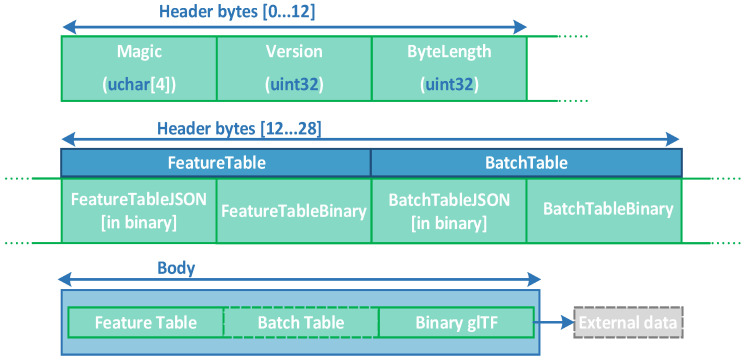
b3dm data structure.

To achieve a conversion from glTF files to 3D Tiles files, the corresponding b3dm tile files must be created. After encapsulating the glTF file and the JSON (JavaScript object notation) file containing the attribute information, the tile data b3dm file is obtained. The file structure is shown in Figure 5. The B3DM file is composed of two main components: the File Head and the File Body. The File Head records essential information, such as the file type, memory, and version. The File Body contains the relevant data, including the number of models, semantic information, and geometric data. Finally, adding the tileset file to the obtained file produces the 3D Tiles target file format.

### 2.3. Dynamic Data Scheduling

**Figure 6:**
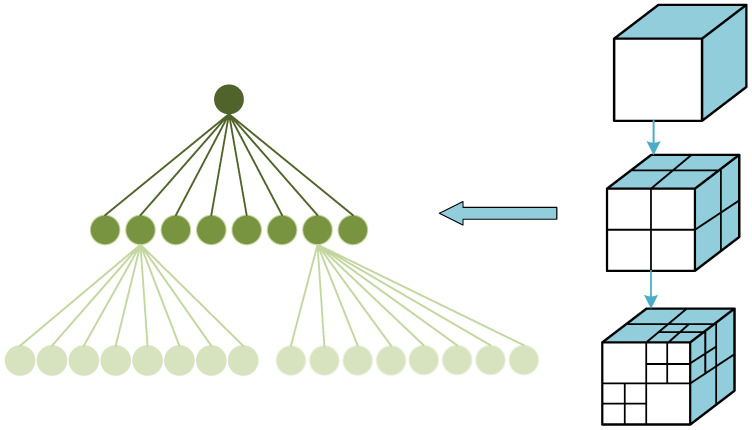
Illustration of octree principle.

Three-dimensional data occupy a large amount of storage space. Loading data by traversing all nodes while restricted by limited network bandwidth and server performance will inevitably impede the rendering speed and frame rate of the data. LOD technology is an efficient solution for smoothly loading massive data in a scene, especially LOD hierarchical loading technology, which determines resource allocation by considering the significance and positional information of the target object in the environment. This reduces the details of secondary target objects and increases the rendering efficiency of the target. Based on this approach, the octree method is adopted to organize three-dimensional data slicing, which is combined with different LOD three-dimensional tiles that are obtained after lightweight processing to achieve a hierarchical structure detail-level subdivision, as shown in Figure 6. 

The octree spatial partitioning structure provides a simple and efficient method for data analysis and processing. By equally dividing cubic space into eight parts, a parent–children relation is created between the unperturbed large cube and its eight partitioned cubes. This operation is applied repeatedly on the eight new cubes until a termination condition is met, resulting in a tree-like structure containing volume elements whereby each node can have either zero or eight child nodes. BIM data are stored within each subdivided area using tiled caches with a range of edge lengths between 100 and 300 m. These caches contain three-dimensional slices with variable resolutions, such as 2048 × 2048, 1024 × 1024, 512 × 512, 256 × 256, 128 × 128, and 64 × 64. To optimize the loading efficiency, a pixel-filtering threshold of two is set to omit sub-objects with fewer than two pixels.

Through the use of an octree indexing structure, different LOD models under different hierarchical structures can be loaded rapidly. During data browsing, users only observe a part of the data, and the remaining data do not affect the final output. Based on this LOD index hierarchy, view frustum culling reduces the rendering data and furthers the improvement of data scheduling efficiency.

**Figure 7:**
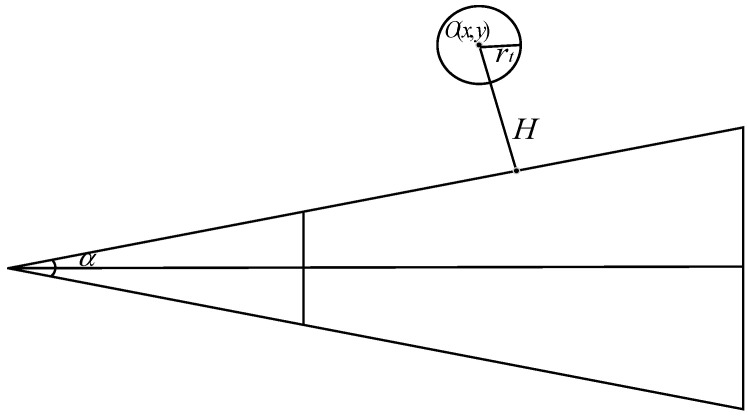
Schematic diagram of frustum culling.

Excluding three-dimensional objects that lie outside of the viewing frustum is known as view frustum culling, and in practice, refers to not loading tile data whose bounding box does not intersect with the viewing frustum. Figure 7 illustrates the implementation principle for a spherical bounding box and viewing frustum. Where point *O* represents the center of the bounding box, (x,y) is the point coordinate, $r_{t}$ is the approximate radius of the bounding box, α is the angle of the viewing frustum, and H is the shortest distance between the viewing frustum boundary and point *O*. Using geometric calculation, it can be obtained that [28]:

(10) $$H=(y-x\times \tan\frac{\alpha }{2})\times \cot\frac{\alpha }{2}.$$

To determine whether to load a node or not, its bounding box radius is compared. If the result is positive, the node is not loaded; otherwise, it is loaded.

**Figure 8:**
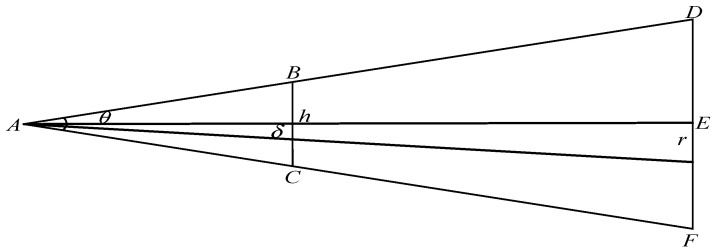
Schematic diagram of screen space error.

As regards models inside the view frustum, a higher resolution is required when the tile data are near the viewer located in the view frustum direction, implying a higher LOD value for the model. Conversely, when the tile data are far from the viewer, lower resolution tile data will suffice, thus yielding a lower LOD value for that specific area. The screen space error determines the specific loading level, with its value calculated using the sum of the geometric error and the view frustum data shown in Figure 8. r represents the geometric error, d represents the distance between the viewpoint and the far clipping plane (segment *AE*), θ represents the angle of the view frustum, h represents the screen height (segment *BC*), and δ corresponds to the screen space error. Based on the perspective projection geometry, we can derive the relationship:

(11) $$\frac{\delta }{r}=\frac{h}{2d\times \tan(\theta /2)}$$

May be defined as:

(12) $$\delta =\frac{hr}{2d\times \tan(\theta /2)}.$$

To decide whether to load the next level of nodes, we compare the screen space error δ with the threshold ε. If δ<ε, the next level of nodes are not loaded; if δ>ε, the next level of nodes are loaded.

## 3. Visualization Applications

**Figure 9:**
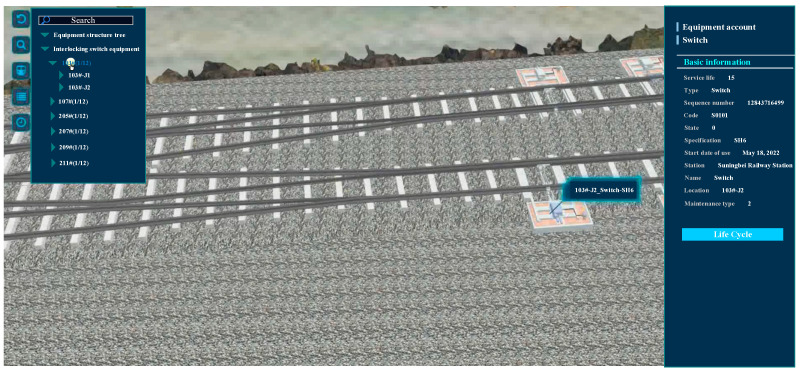
Outdoor equipment display.

**Figure 10:**
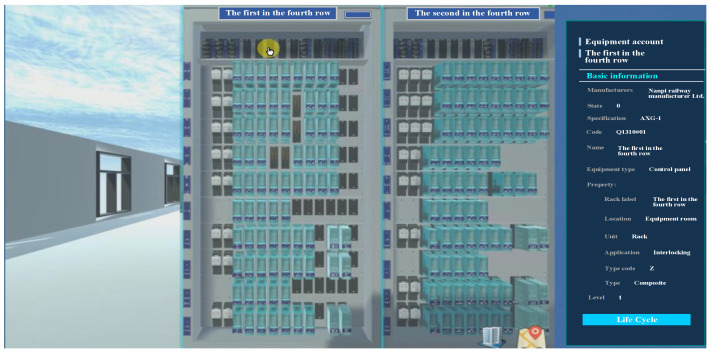
Indoor equipment display.

The railway signal operation and maintenance integrated platform based on BIM + GIS can integrate various types of information regarding railway signal equipment. The most critical aspect entails the collection, processing, analysis, evaluation, and decision-making of alarms regarding monitoring data for the signal equipment on existing lines. As illustrated in Figure 9, the outdoor equipment model has a seamless integration with the map model. The figure presents a comprehensive representation of the outdoor signal equipment used in railway stations. The left column of the figure exhibits a structural tree, which outlines the categorization of eight different types of outdoor signal equipment, such as interlocking switch devices and station track circuit equipment. The right column of the figure displays the relevant account information for each type of equipment, including their service life, equipment type, and serial number. Moreover, Figure 10 provides a detailed presentation of the signal relay combination cabinet’s structure and installation location inside the station building. Specifically, it displays the fourth row and the first and second racks of the signal relay combination cabinet, along with the account information, including manufacturer, status, and specifications, shown on the right side. Through this platform, users can interact with the model and develop a comprehensive understanding of the model and the entire engineering project from macro- to microlevel analysis. This ability satisfies the overall display needs for presenting large files, drawings, and models.

The Railway Signal Operation and Maintenance Integrated Platform based on BIM + GIS enables the direct browsing of station and equipment situations in 3D. With key field searches, staff can locate equipment and access their basic information and maintenance records by clicking on them. Additionally, the platform automatically collects and transmits the equipment’s status in real-time to the monitoring platform via intelligent collection base stations. The comprehensive warning system uses monitoring data to generate tables and line charts, thus performing all-time automated operation monitoring and data processing. The platform displays the alarm status, such as power module status, card status, and system connection status of track circuits, switches, signal machines, and other indoor signal system equipment in a graphical way. By utilizing the collected monitoring data, the platform performs real-time analysis and processing, predicting and alerting potential failures, providing more intuitive and efficient means for staff to conduct daily maintenance and fault handling.

## 4. Verification

To further authenticate the proposed BIM + GIS integration application, a model of an entry signal machine was chosen for testing in this study. The size of the model is 86.9 MB. The experimental hardware consisted of an Intel Core i9-12900K CPU, 64 GB of memory, and an NVIDIA GeForce RTX3080TI GPU. The experimental software comprised the Chrome browser and a Node.js server.

### 4.1. Effect Verification of Model Lightweight Processing

**Figure 11:**
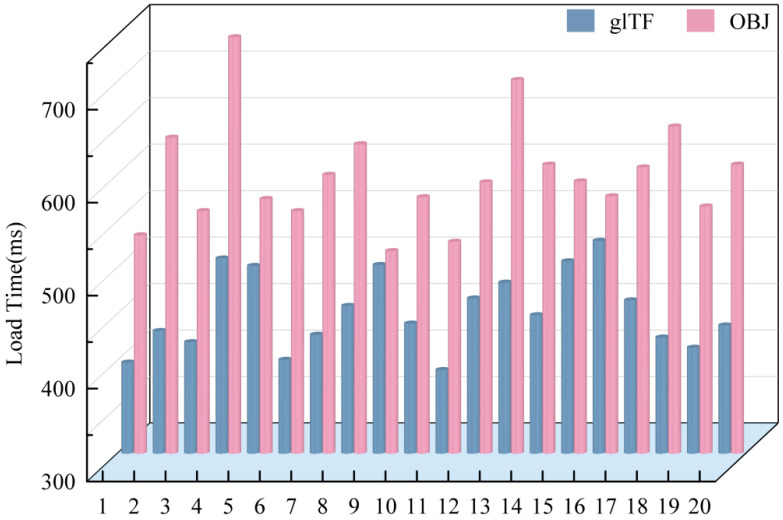
A comparison of loading time for different file formats.

The selection of the appropriate format for a 3D model affects its loading speed on the web. Thus, it is essential to choose a format that is suitable for web loading and rendering. In this paper, we performed loading effect tests with Cesium on 20 groups of data representing 3D models in both glTF and OBJ formats with different data structures. By comparing and analyzing the results, we examine the loading performance of each format. The loading time comparison chart, which is provided in Figure 11, presents our findings.

The chart data reveal that the performance of loading 3D models in the glTF format from the web demonstrates an average loading time of 24.8 s, with a range of 23.2 to 26.1 s when performing 20 loading tests. In contrast, the performance of loading models in the OBJ format from the web displays an average loading time of 27.5 s, with a range of 26.8 to 28.3 s when conducting 20 loading tests.

The analysis and test results indicate that converting original 3D model files into a more structured OBJ format takes longer for Cesium to render the loading process. However, when loading 3D models in glTF format on the web, the loading time is significantly shorter than using other file formats, and the maximum and minimum loading time values are also smaller than those for OBJ format. To enhance the loading and rendering efficiency of BIM models on the web, it is recommended to use the glTF format first and then load and render it through the Cesium engine on the front end. This is because rendering different formats on the web may consume additional performance, while using the glTF format can reduce the extra rendering consumption and effectively diminish the loading time of BIM models.

To obtain a lightweight effect for BIM models and to optimize their rendering and loading, we simplified the mesh information of the BIM model used for the entrance signal machine in order to reduce its data volume. Table 1 shows the results of the model volume comparisons. The comparison results demonstrate that the lightweight processing achieved good simplification while preserving the model’s geometric and material characteristics. Specifically, the significant reduction in the model volume optimizes the network transmission speed.

### 4.2. Data Fusion Visualization

**Figure 12:**
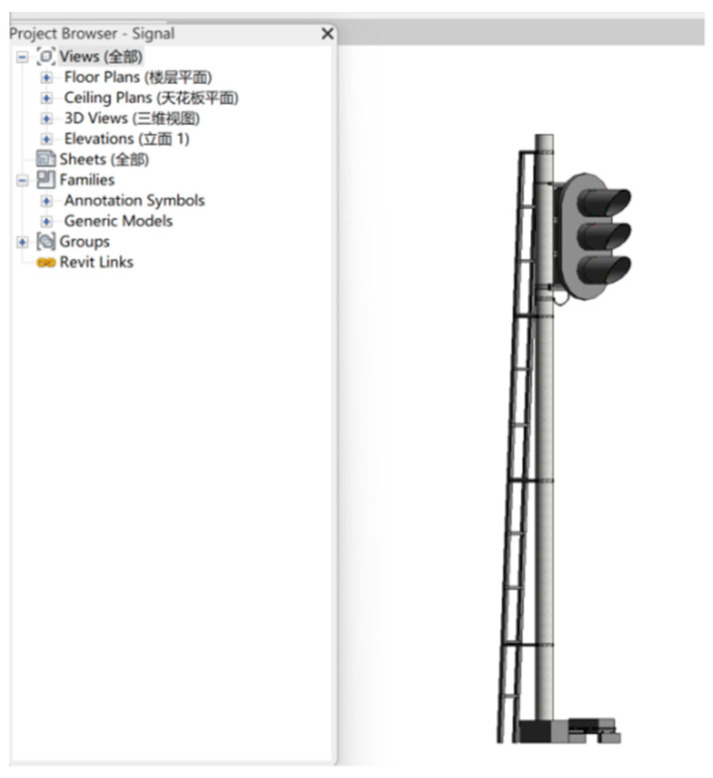
BIM model of the entrance signal machine.

**Figure 13:**
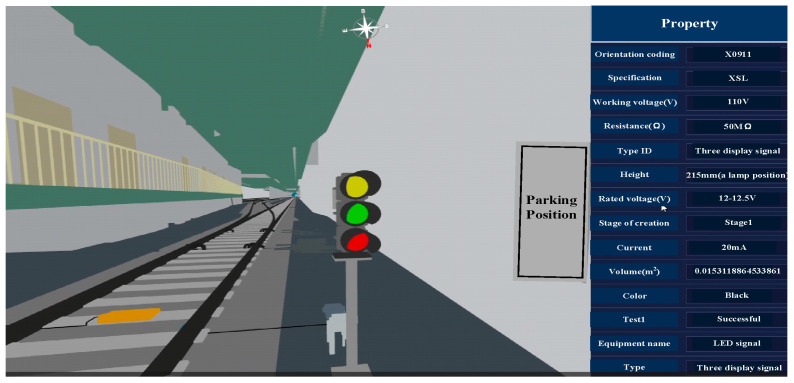
Entrance signal machine model loaded into Cesium engine.

The BIM model for the entrance signal machine represented in Figure 12 was subjected to lightweight processing and processed according to the system data model conversion plan to accomplish the integration of BIM + GIS heterogeneous data sources. The resulting 3D Tiles target format was subsequently loaded into the Cesium engine for rendering, as depicted in Figure 13. These results confirm the successful coordinate transformation and preservation of geometric information. Moreover, a device attribute box will pop up on the right-hand side of the screen as users click on the in-station signal device component with the mouse. The attribute box displays the relevant information for the signal device, including its appearance traits, such as length, width, height, volume, and color; electrical properties, such as operating voltage, current, resistance, etc., and type ID. The presentation of information in this attribute box indicates the distinct independence between geometric components and the retention of component attribute information.

### 4.3. Comparison of Different Data Scheduling Methods

**Figure 14:**
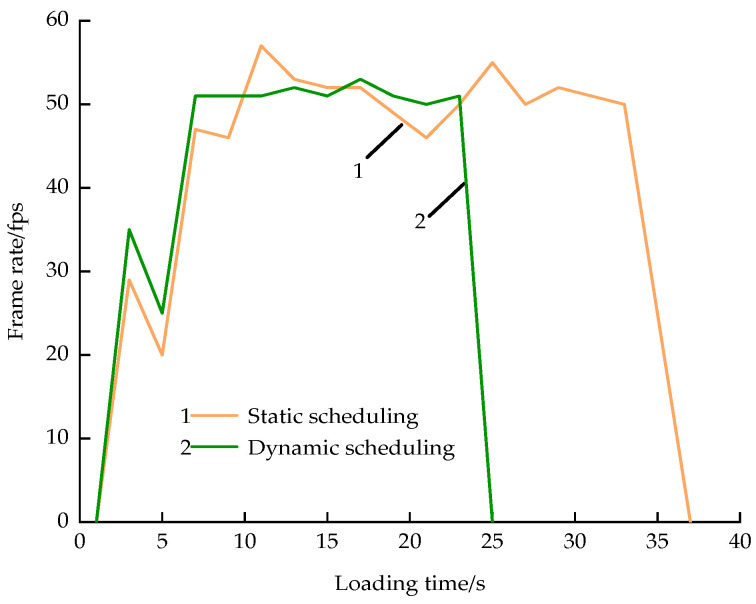
Comparison of model load efficiency.

Following the construction of the b3dm 3D tiles, dynamic scheduling of 3D data was conducted using an octree structure and view-frustum culling algorithm. The performance of the dynamically scheduled data in terms of loading and rendering was compared with that of the statically scheduled data, as demonstrated in Figure 14. The figure illustrates that the frame rate for both loading times remained stable at approximately 50 frames per second (fps). Notwithstanding, dynamic scheduling exhibited greater stability and necessitated a shorter loading time. Without the implementation of an octree structure and view-frustum culling algorithm, the instabilities in the frame rate and extended loading times would be more conspicuous, especially with larger data sizes and when the experimental platform’s efficiency is insufficient. Thus, the application of dynamic scheduling using an octree structure and a view-frustum culling algorithm can significantly enhance the loading and rendering efficiency of the data.

It can be concluded that the model’s entrance signal rendering and loading processes were efficient, achieving the intended outcomes. This is attributed to the clearly defined LOD levels, the independence of geometrical components, and the well-preserved attribute information.

## 5. Conclusions

(1) To address the issue of high complexity and large volume of BIM models, a solution is proposed that is based on simplified mesh information. The solution reduces the amount of data in the model while ensuring that it remains deform-free, enabling faster loading and display.
(2) To address differences in model data resulting from differences in emphasis between BIM and GIS technologies, the spatial index structure of the model data was designed, the component coordinate system was transformed, and the LOD levels were divided reasonably. This approach ensured the fast rendering and loading of BIM data in the 3D Tiles file format on the Cesium engine, resulting in the successful integration of BIM and GIS applications.
(3) An octree index structure was designed and combined with a view-frustum culling algorithm to dynamically schedule massive model data, effectively improving the rendering efficiency of models and reducing the server’s burden.
(4) In the context of the railway industry’s digital transformation, the BIM model has been integrated into the WebGIS platform. This integration allows for real-time analysis and the processing of monitoring data related to railway signaling equipment operation. The integration also provides intelligent warnings in instances of railway signaling equipment faults. Subsequently, this technological advancement expands the application of BIM technology within the railway field while significantly enhancing the level of equipment operation and maintenance.
(5) The method presented in this article only performs lightweight processing of the surface, i.e., geometric information, of the BIM model, excluding the processing of any non-geometric information. It is necessary to further study lightweight integration theoretical methods and optimize visual management, given the increasing volume of railway industry models and health data. These efforts will better address the new challenges presented by the railway’s ongoing digitalization.

## Figures and Tables

**Table 1:** Model volume comparison.

Document Type	File Memory/MB
RVT	86.9
glTF(LOD5)	68.4
glTF(LOD4)	52.1
glTF(LOD3)	48.2
glTF(LOD2)	33.8

## Data Availability

Not applicable.
